# Exogenous mitochondrial transplantation improves survival and neurological outcomes after resuscitation from cardiac arrest

**DOI:** 10.1186/s12916-023-02759-0

**Published:** 2023-03-16

**Authors:** Kei Hayashida, Ryosuke Takegawa, Yusuke Endo, Tai Yin, Rishabh C. Choudhary, Tomoaki Aoki, Mitsuaki Nishikimi, Atsushi Murao, Eriko Nakamura, Muhammad Shoaib, Cyrus Kuschner, Santiago J. Miyara, Junhwan Kim, Koichiro Shinozaki, Ping Wang, Lance B. Becker

**Affiliations:** 1 Laboratory for Critical Care Physiology, The Feinstein Institutes for Medical Research, Northwell Health, Manhasset, NY USA; 2 Center for Immunology and Inflammation, The Feinstein Institutes for Medical Research, Northwell Health, Manhasset, NY USA

**Keywords:** Mitochondrial transplantation, Cardiac arrest, Ischemia and reperfusion, Mitochondria

## Abstract

**Background:**

Mitochondrial transplantation (MTx) is an emerging but poorly understood technology with the potential to mitigate severe ischemia–reperfusion injuries after cardiac arrest (CA). To address critical gaps in the current knowledge, we test the hypothesis that MTx can improve outcomes after CA resuscitation.

**Methods:**

This study consists of both in vitro and in vivo studies. We initially examined the migration of exogenous mitochondria into primary neural cell culture in vitro. Exogenous mitochondria extracted from the brain and muscle tissues of donor rats and endogenous mitochondria in the neural cells were separately labeled before co-culture. After a period of 24 h following co-culture, mitochondrial transfer was observed using microscopy. In vitro adenosine triphosphate (ATP) contents were assessed between freshly isolated and frozen-thawed mitochondria to compare their effects on survival. Our main study was an in vivo rat model of CA in which rats were subjected to 10 min of asphyxial CA followed by resuscitation. At the time of achieving successful resuscitation, rats were randomly assigned into one of three groups of intravenous injections: vehicle, frozen-thawed, or fresh viable mitochondria. During 72 h post-CA, the therapeutic efficacy of MTx was assessed by comparison of survival rates. The persistence of labeled donor mitochondria within critical organs of recipient animals 24 h post-CA was visualized via microscopy.

**Results:**

The donated mitochondria were successfully taken up into cultured neural cells. Transferred exogenous mitochondria co-localized with endogenous mitochondria inside neural cells. ATP content in fresh mitochondria was approximately four times higher than in frozen-thawed mitochondria. In the in vivo survival study, freshly isolated functional mitochondria, but not frozen-thawed mitochondria, significantly increased 72-h survival from 55 to 91% (*P* = 0.048 vs. vehicle). The beneficial effects on survival were associated with improvements in rapid recovery of arterial lactate and glucose levels, cerebral microcirculation, lung edema, and neurological function. Labeled mitochondria were observed inside the vital organs of the surviving rats 24 h post-CA.

**Conclusions:**

MTx performed immediately after resuscitation improved survival and neurological recovery in post-CA rats. These results provide a foundation for future studies to promote the development of MTx as a novel therapeutic strategy to save lives currently lost after CA.

**Supplementary Information:**

The online version contains supplementary material available at 10.1186/s12916-023-02759-0.

## Background

Mitochondrial transplantation (MTx) is an emerging technology with the potential to improve function in cells damaged by ischemia and reperfusion (I/R). During cardiac arrest (CA), ischemic injury rapidly depletes cellular adenosine triphosphate (ATP), generates free radicals, and dysregulates the ionic control of sodium and calcium. These mechanisms trigger additional signaling cascades that can worsen during reperfusion, leading to profound mitochondrial dysfunction and death [1–4]. Fortunately, injured mitochondria are capable of self-repair and cellular protection through fission, fusion, mitophagy, and the recently described mechanism of intercellular mitochondrial transfer [5, 6]. The discovery that mitochondria can migrate from cell to cell suggests the possibility of transplanting healthy donor mitochondria into cells with injured mitochondria [7–9].

A number of studies have identified improved outcomes following MTx in the fields of cardiac injury [10–17] and stroke [6, 18]. Mitochondria have been directly injected into target tissues or delivered by simple intravenous infusion [18]. However, the extent to which mitochondria are taken up by neurons, cardiac myocytes, and other organs is not known. It is not known how long donor mitochondria persist in tissues as healthy (respiring) mitochondria or whether they simply provide short-lived biologically active mitochondrial particles. Furthermore, MTx has not been studied in I/R injury following CA.

CA afflicts over 500,000 individuals in the USA each year [19]. CA halts all circulation throughout the body, resulting in whole-body ischemia which is fatal if left untreated. Despite advances in the resuscitation and post-arrest management of patients who suffer from CA, mortality exceeds 90%, and of those who survive, many exhibit prolonged neurological deficits and cardiovascular complications [20–22]. At present, there are few effective drugs or other therapies that can meaningfully improve these poor outcomes [23]. CA, as well as other ischemic emergencies, remains a significant public health challenge.

Our study tests the hypothesis that MTx can improve the outcomes after resuscitation from a severe ischemic injury such as a CA. We focus on three important questions regarding MTx: (1) Do exogenous allogeneic mitochondria, extracted from the brain or muscle, successfully enter neural cells in culture? (2) Does the intravenous infusion of fresh mitochondria delivered immediately after CA change survival rates and other physiological indicators of I/R injury in an animal model? (3) Do transplanted mitochondria persist in tissues 24 h after CA? We answer these questions via a sequence of experimental studies that provide a foundation to further evaluate the role of MTx in mitigating multiple organ injury and mortality after resuscitation from CA.

## Methods

### Cell culture

To determine whether exogenous donor mitochondria can be taken up into neurons growing in culture, we co-cultured exogenous mitochondria extracted from the brain or muscle tissues of donor rats with neural cell cultures. Both donor mitochondria and recipient neural cell cultures were derived from male Sprague–Dawley rats (12 weeks old; Charles River Laboratories, Wilmington, MA, USA). Neural cells were isolated using the Adult Brain Dissociation kit (Miltenyi Biotec, Inc., Somerville, MA, USA). Cells were seeded at a density of 1 × 10^5^ cells/cm^2^ onto glass coverslips coated with poly-d-lysine (0.1 mg/mL). The endogenous (native) mitochondria of the cells were separately labeled 24 h before mitochondrial transfer with MitoTracker dye (Thermo Fisher Scientific, Waltham, MA, USA) according to the manufacturer’s instructions. Briefly, the cells were suspended in a prewarmed (37 °C) staining solution containing the MitoTracker Green probe (300 nM) and incubated for 30 min in a standard medium under appropriate growth conditions. After staining, the cells were washed twice with phosphate-buffered saline (PBS) and resuspended in a fresh medium.

To visualize mitochondrial transfer, neural cells in which endogenous mitochondria were stained green (see above) were co-cultured with donated exogenous mitochondria (red) in a standard culture medium for 24 h, and cells were observed using an LSM 880 confocal imaging system (Carl Zeiss Meditec AG, Jena, Germany).

### Mitochondrial isolation from brain tissue

For MTx experiments in the cell culture, brain tissues in mitochondrial isolation buffer [210 mM d-mannitol, 70 mM sucrose, 5 mM HEPES, 1 mM EGTA, and 0.5% (w/v) fatty acid-free bovine serum albumin (BSA), adjusted pH to 7.2 with KOH] were disrupted by 30 strokes at 500 rpm in a homogenizer, after which the homogenate was centrifuged at 800* g* for 10 min at 4 °C in a swing-out rotor. The supernatant was then centrifuged at 12,000* g* for 10 min at 4 °C to create a pellet containing the mitochondria. After removal of the supernatant, the pellet was washed twice with mitochondrial isolation buffer, and the pellet was resuspended in prewarmed (37 °C) staining solution with PBS containing the MitoTracker Deep Red probe (300 nM) and incubated for 30 min. After the removal of the staining solution, the labeled mitochondria were washed twice with PBS. Mitochondria were quantified by determining the protein concentration using the Bradford assay (Pierce, Rockford, IL, USA) and kept on ice until transplantation. Mitochondria (0.01 mg/mL, final concentration) resuspended in 500 μL of fresh prewarmed medium were immediately used for mitochondrial transfer.

### Mitochondrial isolation from pectoral muscles

Muscle-derived mitochondria from rats were used both for MTx experiments (A) in cell culture and (B) as donor mitochondria used by infusion into an in vivo rat as outlined below in our CA model protocol. Mitochondria were isolated from a 6-mm piece of healthy pectoralis major muscle tissue from a rat using a rapid mitochondrial isolation method, as previously described [24]. This method using an automated homogenizer and different filtrations developed for clinical use when speed was essential as the full procedure may be completed in 30 min was recently reported by McCully et al. [8, 10, 24]. Briefly, immediately after obtaining the muscle using a 6-mm biopsy punch, the tissue was minced in cold homogenized buffer [300 mM sucrose, 10 mM K-HEPES, and 1 mM K-EGTA (pH 7.2)] at 4 °C and homogenized using an automated homogenizer (gentleMACS dissociator; Miltenyi Biotec Inc., San Diego, CA, USA). The homogenate was then subjected to digestion for 10 min with subtilisin A on ice (protease from *Bacillus licheniformis*; Sigma-Aldrich, St. Louis, MO, USA), and the digested homogenate with 0.49% fatty-acid free BSA was filtered through a series of disposable sterile mesh filters. The filtrate was centrifuged at 9000* g* for 10 min at 4 °C, and the final pellet was resuspended in 0.5 mL of a cold respiration buffer [250 mM sucrose, 2 mM KH_2_PO_4_, 10 mM MgCl_2_, 20 mM K-HEPES (pH 7.2), 0.5 mM K-EGTA (pH 8.0)]. The yield of mitochondrial particles obtained using a 6-mm biopsy tissue sample was reported to be approximately 1 × 10^10^ mitochondria, which provided sufficient mitochondria for infusion, as well as quality assurance and quality control assessment [8, 10, 24]. Previous studies have consistently demonstrated the viability and functionality of mitochondria isolated from the skeletal muscle using this method [10, 16, 24, 25]. Isolated mitochondria were used immediately for intravenous infusion as fresh donor mitochondria or were frozen and stored at − 80 °C for over 2 weeks until subsequent use as frozen-thawed mitochondria.

### Measurements of ATP content in isolated mitochondria

ATP contents were determined in (a) respiration buffer as a negative control, (b) frozen-thawed, and (c) freshly isolated mitochondria using a luminescent assay kit (ATPlite, PerkinElmer, MA) according to the manufacturer’s instructions. A total of 10 µL of mitochondrial particles from the prepared samples or respiration buffer were added to each well of a white, opaque bottom, 96-well plate. After measuring luminescence, ATP concentration in each well was calculated using the standard curve obtained from ATP standard stock solution.

### Flow cytometry analysis of JC1 assay for isolated mitochondria

The mitochondrial membrane potential (ΔψM) was evaluated by MitoProbe™ JC1 (5′,6,6′-tetrachloro-1,1′,3,3′-tetraethylbenzimidazolylcarbocyanine iodide) Assay Kit (Thermo Fisher Scientific, Waltham, MA) using a BD FAC Symphony flow cytometer (BD Biosciences, San Jose, CA). JC1 exhibits potential-dependent accumulation (J-aggregates) in mitochondria, indicated by a fluorescence emission shift from green (~ 529 nm) to red (~ 590 nm). Therefore, ΔψM can be assessed by an increase in the red fluorescence J-aggregates. After isolation, mitochondrial suspension in 1 mL respiration buffer was mixed with 10 μL of 200 μM JC1 (2 μM final concentration) with or without 1 μL of 50 mM carbonyl cyanide 3-chlorophenylhydrazone (CCCP, 50 μM final concentration). CCCP is a well-established mitochondrial membrane potential disrupter. After incubation at 37 °C for 30 min, the suspension was centrifuged at 9000* g* for 10 min at 4 °C. The pellets were washed once by adding 1 mL PBS and centrifuged. The pellets were resuspended in 500 µL fresh respiration buffer. Unstained samples or size reference beads (Spherotech, Inc., Lake Forest, IL) were used to establish a proper mitochondrial size and voltage setting. The acquisition for JC1 Red-positive events was performed on 100,000 events. The percentages of the JC1 Red fluorescence J-aggregates were measured as ΔψM in the freshly isolated mitochondria, frozen-thawed mitochondria, and a subgroup of frozen-thawed mitochondria which was treated with a membrane potential disrupter CCCP as the lowest ΔψM control. Unstained mitochondria were used as a negative control. The data were analyzed with the FlowJo software (Tree Star, Ashland, OR, USA).

### Animal care and surgical preparation

Adult male Sprague–Dawley rats (400‒545 g, 12‒16 weeks old; Charles River Laboratories) were used in this in vivo study. The animals were housed in a rodent facility under a 12:12-h light/dark cycle with ad libitum access to food and water. The rats were intubated with a 14-gauge plastic catheter (Surflo; Terumo Medical Corporation, Somerset, NJ, USA) under anesthesia with 4% isoflurane (Isosthesia; Butler–Schein AHS, Dublin, OH, USA), mechanically ventilated, and surgically prepared under anesthesia (2% isoflurane). Before the surgical procedure, surgical sites were cleaned with povidone-iodine and then covered with a sterile, self-adhesive, transparent, povidone-treated surgical blanket. All surgical procedures were conducted using sterile equipment and performed by the investigators blinded to the experimental groups. End-tidal carbon dioxide was maintained at 40 ± 5 mmHg during the experiment by adjusting the respiratory rate (RR) and tidal volume (TV), with these settings adjusted within the range of 40/min to 50/min for the RR and 3.5 to 5.0 mL of TV. Microcatheters (PE-50; Becton Dickinson, Franklin Lakes, NJ, USA) were inserted into the left femoral artery and left femoral vein to monitor blood pressure and infuse drugs and donor mitochondria, respectively. Heparin (300 U) was injected into the femoral vein. The esophageal temperature was maintained at 37.0 ± 0.5 °C using a thermostatically regulated heating pad and heating lamp during the experiment. Blood pressure and needle-probe electrocardiogram-monitoring data were recorded and analyzed using a personal computer-based data-acquisition system.

### Rat cardiac arrest protocol

All animal studies were performed using protocols approved by the Institutional Animal Care and Use Committee at our institution and in accordance with National Institutes of Health guidelines. Rats were subjected to CA and cardiopulmonary resuscitation, as previously described [26, 27], with minor modifications. Briefly, prior to the induction of asphyxia, rats were mechanically ventilated with a fraction of inspired O_2_ (F_I_O_2_; 0.3), and anesthesia was maintained with 2% isoflurane during surgical procedures. Asphyxia was induced by intravenous vecuronium bromide (2 mg/kg), followed by switching off the ventilator and discontinuation of isoflurane. CA was defined as a mean arterial pressure of < 20 mmHg. At 10 min after induction of asphyxia, mechanical ventilation was restarted at an F_I_O_2_ of 1.0, and manual chest compressions were performed at a rate of 300/min by a single investigator blinded to the experimental groups. At 30 s after beginning chest compressions, a 20-μg/kg bolus of epinephrine was administered, and chest compressions were continued until successful resuscitation, which was defined as the return of supraventricular rhythm with a mean arterial pressure of > 60 mmHg for 10 s. Rats were mechanically ventilated with an F_I_O_2_ of 1.0 for the first 10 min after resuscitation, after which F_I_O_2_ was reduced to 0.3, followed by disconnection from the mechanical ventilator and extubation at 2 h post-CA. Arterial blood pressure, electrocardiogram recordings, and esophageal temperature were monitored for 2 h. Arterial blood samples for blood gas and lactate analyses were obtained at baseline and 15- and 120-min post-CA. No additional inotropic agent was administered. After a recovery period of 2 h, the animals were weaned from the ventilator, all vascular catheters and tracheal tubes were removed, and surgical wounds were sutured. The rats were then returned to their cages with easily accessible food and water, and observed in a rodent facility with a controlled room temperature of 22 °C. Buprenorphine XR (0.2 mL; 0.26 mg) was subcutaneously injected once to relieve any pain due to incisions in all animals during the recovery period. The survival time after CA was recorded for up to 72 h.

### Assessment of neurological function

Neurological function score (NFS) was evaluated by a blinded investigator at 24, 48, and 72 h post-CA using a previously reported neurofunctional scoring system [27]. With this score, neurologically normal animals would receive a score of 500, while dead or brain-dead rats were scored at 0 points.

### Echocardiography

We assessed left ventricular ejection fraction (LVEF) at baseline and 2 h post-CA using echocardiography. Transthoracic closed-chest echocardiography was performed by a single blinded investigator using a 12–4 MHz probe (S12-4 sector array transducer; Philips, Amsterdam, The Netherlands), and all measurements were averaged over three cardiac cycles.

### Assessment of 72-h lung injury

The lung wet-to-dry weight ratio (W/D) was used as an index of pulmonary edema formation. The left lower lobe was removed at 72 h post-CA, weighed immediately after removal (wet weight) and again after drying in an oven at 37 °C for 7 days (dry weight). Lung W/D was calculated as the ratio of wet weight to dry weight.

### Mitochondrial infusion immediately after CA

Animals subjected to CA were block-randomized into one of three groups of interventions that were administered immediately after animals achieved successful resuscitation: (a) infusion of the respiration buffer with 0.49% BSA (vehicle group; *n* = 11), (b) infusion of nonfunctional frozen-thawed mitochondria (frozen-thawed-mito group; *n* = 11), or (c) infusion of fresh viable mitochondria (fresh-mito group; *n* = 11). The process of freezing and thawing leads to widespread disruption of mitochondrial outer membrane integrity and suppresses electron-transport chain activity through the loss of cytochrome c from the inter-membrane space [28]. For that reason, we used frozen-thawed mitochondria as an additional control group to maintain similar amounts of mitochondrial protein, lipids, DNA, RNA, and other macromolecules, as infused into the animals treated with freshly isolated mitochondria. These disrupted frozen-thawed mitochondria contain similar quantities of biological molecules but do not have viability and respiratory competence.

### Measurements of gene expressions

RNA isolation, reverse transcription, and real-time PCR analysis were performed on brain and spleen tissues harvested at 72 h post-CA resuscitation and MTx according to the manufacturer’s instructions. Total RNA was extracted using TRIzol Reagent (Sigma-Aldrich, USA) and reverse transcribed using SuperScript IV VILO™ Master Mix with ezDNase Enzyme (Thermo Fisher, USA). Real-time PCR was performed using TaqMan Fast Advanced Master Mix (Thermo Fisher, USA) on the LightCycler 480 system (Roche Diagnostics). The primers used are dynamin-related protein 1 (*Drp1*, Rn00586466_m1), mitochondrial fission 1 protein (*Fis1*, Rn01480911_m1), optic atrophy-1 (*Opa1*, Rn00592200_m1), Mitofusin-1 (*Mfn-1*, Rn00594496_m1), and Mitofusin-2 (*Mfn-2*, Rn00500120_m1).

### Measurement of cytochrome c oxidase activity

To measure cytochrome c oxidase (COX) activity in tissues, the brain and spleen were obtained from sham-operated rats and surviving rats at 72 h post-CA in the vehicle, frozen-thawed, or fresh-mito groups. The COX activity in tissue homogenates was measured using the Cytochrome C oxidase Kit (Abcam, ab239711) according to the manufacturer’s instructions.

### Brain perfusion measured with laser speckle flowmetry and image processing

In a separate set of experiments aimed at determining the impacts of MTx on brain perfusion during the acute phase post-CA, we monitored relative cerebral blood flow (rCBF) for the first 2 h after CA. Laser speckle imaging of the brain was performed using the full-field laser perfusion imager RFLS III system according to the manufacturer’s instructions (RWD Life Science Co., Ltd., Guangdong, China), as previously described [29]. A midline scalp incision was made to expose the skull for imaging. The skull over the left cortical surface was thinned using a dental drill, ensuring that the dura remained intact. The imager was positioned directly above the surface of the thinned skull. Continuous image acquisition (time constant, 1 s; camera exposure time, 5 ms; laser intensity, 100 mA; resolution, 2048 × 2048) started at the pre-CA baseline and continued until 2 h post-CA. The vessels were recognized according to anatomical characteristics, and then three regions of interest (ROIs) were selected at the pre-CA baseline. The ROIs included the area over the left superior cerebral vein and two capillary areas of the left cortical surface between the superior cerebral veins. The signal intensities of perfusion were calculated at each time point using the laser speckle imaging system software and normalized against the baseline [29]. The means of rCBF values at the three ROIs were compared between the groups.

### Confocal fluorescence microscopy

In a separate set of experiments, rats subjected to CA were used to ascertain the uptake and persistence after 1 and 24 h of labeled mitochondrial in vital organs using a confocal microscope. The freshly isolated mitochondria were labeled with MitoTracker Deep Red immediately after isolation, and the vehicle or the labeled mitochondria were infused upon resuscitation from CA. At 1 or 24 h post-CA, animals were euthanized, and the brain, heart, lung, kidney, liver, and spleen were harvested and fixed with 4% paraformaldehyde solution. Sections were mounted with mounting medium containing 4,6-diamidino-2-phenylindole (DAPI) (Vector Laboratories, Burlingame, CA, USA) and observed using an LSM 880 confocal imaging system (Carl Zeiss Meditec AG, Jena, Germany).

### Statistical analysis

Data represent the mean ± standard deviation. Neurological function scores were compared using a Kruskal–Wallis test, followed by Dunn’s multiple comparison test. Continuous data were analyzed by one-way analysis of variance (ANOVA) with Šidák’s correction for post hoc comparisons between multiple experimental groups. Hemodynamic, body weight changes, laboratory, and rCBF data were examined using a mixed-effects model for repeated-measures analyses, followed by ANOVA with Šidák’s correction for post hoc comparisons. For the in vivo survival study, we performed a power analysis to calculate the sample size necessary to achieve a reliable measurement of the effect. As the mean survival rate at 3 days after CA was expected as 40% in the vehicle group and 85% in the freshly isolated mitochondria group, we anticipated that 11 rats per group were required in each survival study (*α* = 0.05, *β* = 0.2 [power = 80%], two-sided). All data are included (no outlier values or animals were excluded from the study). Kaplan–Meier analysis using the Gehan–Breslow–Wilcoxon test was used to calculate the survival rates between groups. A *P* < 0.05 was considered statistically significant. GraphPad Prism (v.9.2.0; GraphPad Software Inc., La Jolla, CA, USA) was used for all statistical analyses.

## Results

### Isolated brain- and muscle-derived rat mitochondria delivered extracellularly in culture media are taken up by cultured neural cells in vitro

**Fig. 1:**
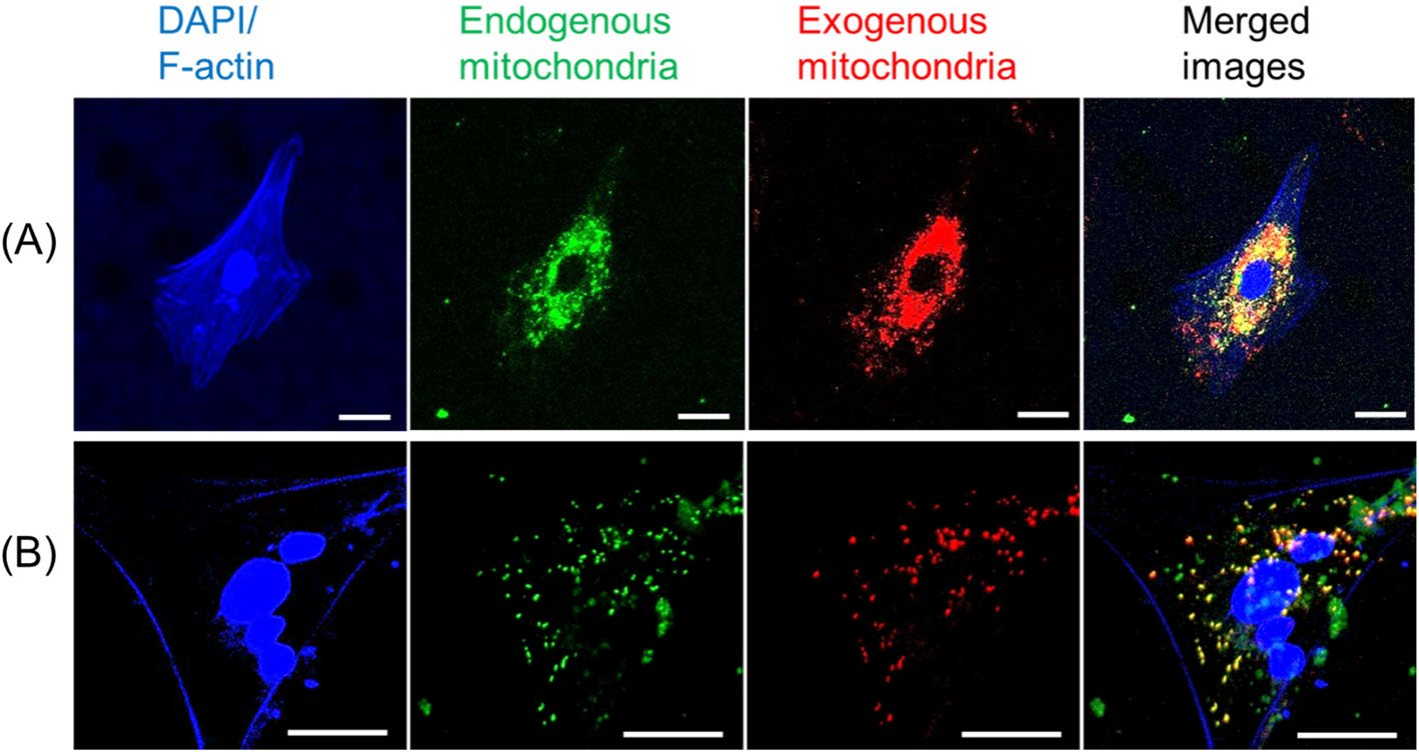
Transfer of exogenous brain- and muscle-derived mitochondria into neural cell cultures. Representative images of exogenous mitochondria stained with MitoTracker Deep Red and co-cultured with brain cells, whose endogenous mitochondria were stained with MitoTracker Green. The exogenous mitochondria (red) were extracted from **A** the brain or **B** the pectoral muscle of the donor rat. Scale bar indicates 20 µm

After tissue isolation, the exogenous donor mitochondria were stained with MitoTracker Deep Red and then co-cultured with neural cells, whose endogenous mitochondria are stained with MitoTracker Green. Exogenous brain mitochondria were taken up into neural cells by simple co-culture for 24 h (Fig. 1A). Transferred exogenous mitochondria (red) co-localized with endogenous mitochondria (green) from neural cells, indicating the movement of exogenous mitochondria inside the cells, as evidenced by merged yellow staining. Additionally, we observed exogenous muscle-derived mitochondrial transfer into neural cells (Fig. 1B). These results suggest that exogenous brain- and muscle-derived mitochondria can be efficiently transferred into neural cells.

### Transplantation of freshly isolated mitochondria improved survival and neurological recovery in post-CA rats

**Fig. 2:**
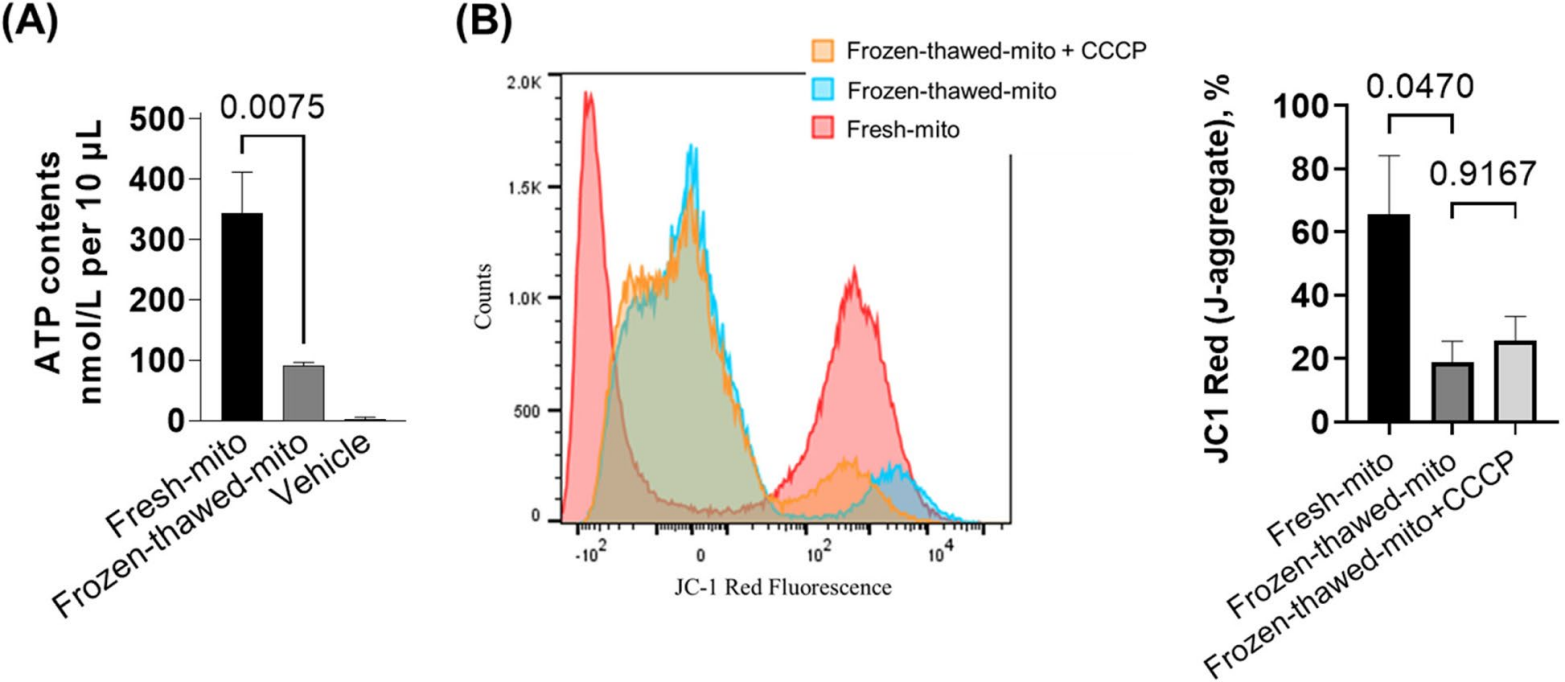
Freshly isolated mitochondria are more functional than frozen-thawed mitochondria. **A** Mitochondrial ATP contents in vehicle, frozen-thawed mitochondria, and fresh mitochondria immediately after mitochondrial isolation. One-way analysis of variance (ANOVA) with Šidák’s correction for post hoc comparisons was used. *n* = 3–4 per group. **B** Flow cytometry analysis of the mitochondrial membrane potential of the isolated mitochondria stained with JC-1 verified that the percentage of J-aggregates was markedly higher in the fresh-mito group than in the frozen-thawed-mito group. Carbonyl cyanide 3-chlorophenylhydrazone (CCCP) did not alter the ΔψM in the frozen-thawed-mito group. The one-way ANOVA with Šidák’s correction for post hoc comparisons was used. *n* = 4 per group

Our most significant finding was a dramatic increase in neurologically intact survival when post-CA rats were treated with infused freshly isolated mitochondria compared to the two control conditions. The three groups of 11 animals each consisted of the following: (1) a simple vehicle treatment as the negative control (respiration buffer with 0.49% BSA), (2) an additional negative control of frozen-thawed nonfunctional mitochondria, and (3) our MTx intervention group that received freshly isolated donor mitochondria. Figure 2A illustrates that freshly isolated mitochondria are functional in comparison with frozen-thawed mitochondria. As expected, ATP content in freshly isolated mitochondria was approximately four times higher than in frozen-thawed mitochondria. The flow cytometry analyses confirmed that the ΔψM was markedly higher in the fresh-mito group compared to the frozen-thawed-mito group (65.60 ± 18.50, 19.06 ± 6.28, *P* = 0.047). CCCP did not alter the ΔψM in the frozen-thawed-mito group (Fig. 2B). These observations suggest that freshly isolated mitochondria have markedly higher ΔψM than frozen-thawed mitochondria and that the frozen-thawed process can be sufficient to disrupt the membrane potential.

**Fig. 3:**
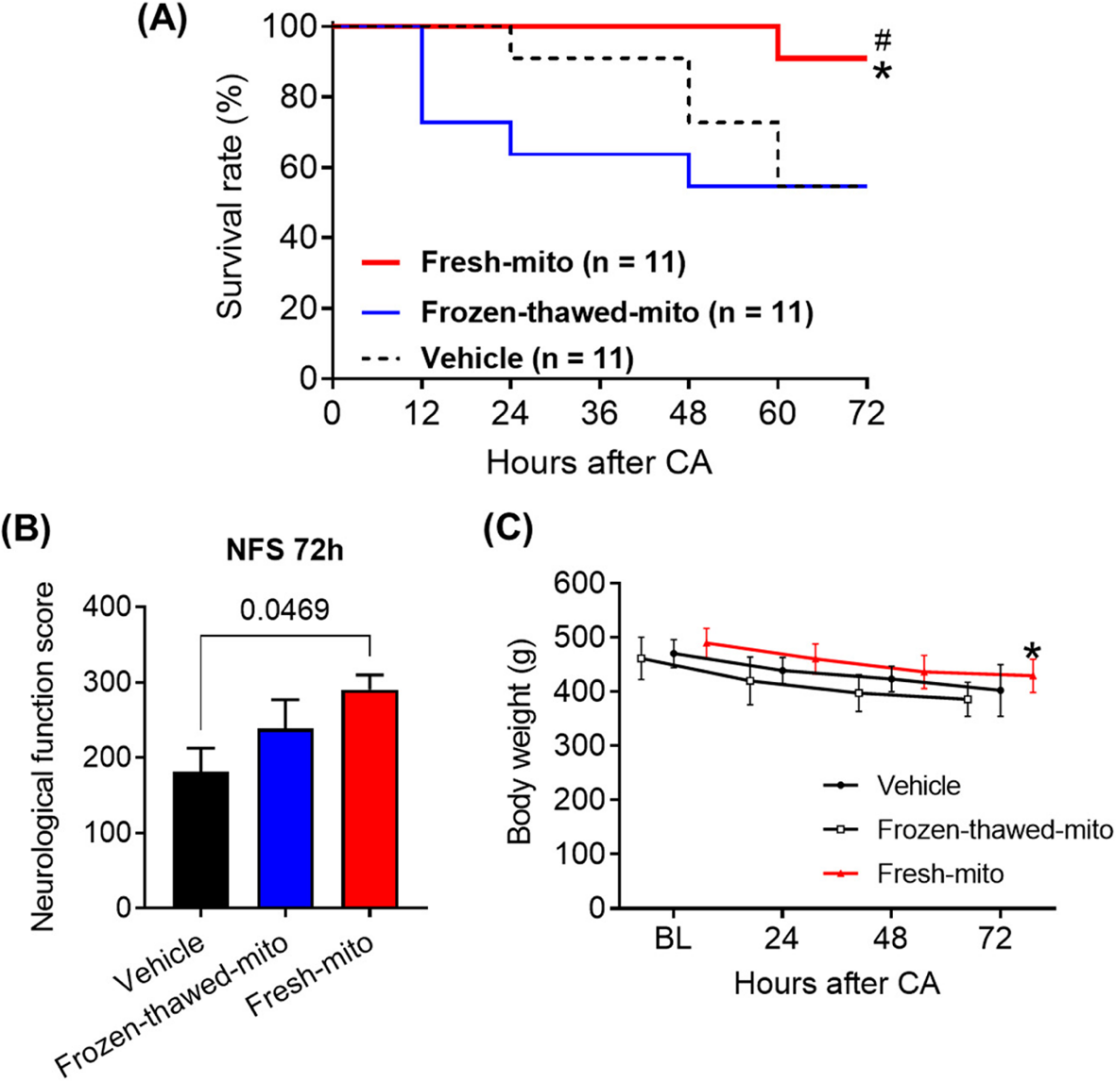
Mitochondrial transplantation using freshly isolated mitochondria improves 72-h neurological function and survival after cardiac arrest and resuscitation. **A** Survival rates during the first 72 h after cardiac arrest (CA) and resuscitation. *n* = 11 per group. *P = 0.048 vs. vehicle group; ^#^*P* = 0.038 vs. frozen-thawed-mito group. Kaplan–Meier analysis using the Gehan–Breslow–Wilcoxon test was used. **B** Neurological functional scores (NFS) at 72 h post-CA in surviving animals in the vehicle, frozen-thawed mitochondria, or freshly isolated mitochondria groups. A score of 0 indicates brain death or dead rats. Dead animals were excluded from the analyses. The Kruskal–Wallis test followed by Dunn’s multiple comparison test was used. **C** Daily changes in body weight in post-CA animals treated with vehicle, frozen-thawed mitochondria, or fresh mitochondria. A mixed-effects model for repeated-measures analyses, followed by one-way ANOVA with Šidák’s correction for post hoc comparisons was used. *P = 0.044 vs. vehicle group. Data represent the mean ± standard deviation

In an in vivo rat model of CA, no differences were observed in baseline characteristics and perioperative hemodynamic parameters between the vehicle, frozen-thawed, and fresh-mito groups (Table 1). The 72-h survival rates in both the vehicle and frozen-thawed-mito groups were 54.5% (6 of 11 rats for both groups). By contrast, animals receiving intravenous injections of fresh mitochondria demonstrated significantly improved 72-h survival rates of 90.9% (10 of 11 rats; *P* = 0.048 vs. vehicle; *P* = 0.038 vs. frozen-thawed-mito) (Fig. 3A). Additionally, conditional on survival, the NFS was significantly higher in the fresh-mito group than in the vehicle group at 72-h post-CA (*P* = 0.047) (Fig. 3B). We also assessed the measures of weight maintenance in surviving rats 72 h after resuscitation. Body weights in the fresh-mito group were significantly higher than those in the frozen-thawed-mito group at 72 h post-CA (Fig. 3C).

**Table 1:** Baseline and experimental parameters in the vehicle, frozen-thawed mitochondria, and freshly isolated mitochondria groups

	Vehicle (*n* = 11)	Frozen-thawed-mito (*n* = 11)	Fresh-mito (*n* = 11)
Weight, g	471 ± 25.9	462 ± 39.2	490 ± 27.2
Heart rate at baseline, bpm	298 ± 41	312 ± 43	289 ± 45
MAP at baseline, mmHg	100 ± 23	100 ± 19	98 ± 25
Time to cardiac arrest, s	205 ± 28	213 ± 80	205 ± 23
Time to resuscitation, s	85 ± 16	76 ± 21	78 ± 13

Values represent mean ± standard deviation

*MAP* mean arterial pressure, *frozen-thawed-mito* frozen-thawed mitochondria, *fresh-mito* freshly isolated mitochondria

**Fig. 4:**
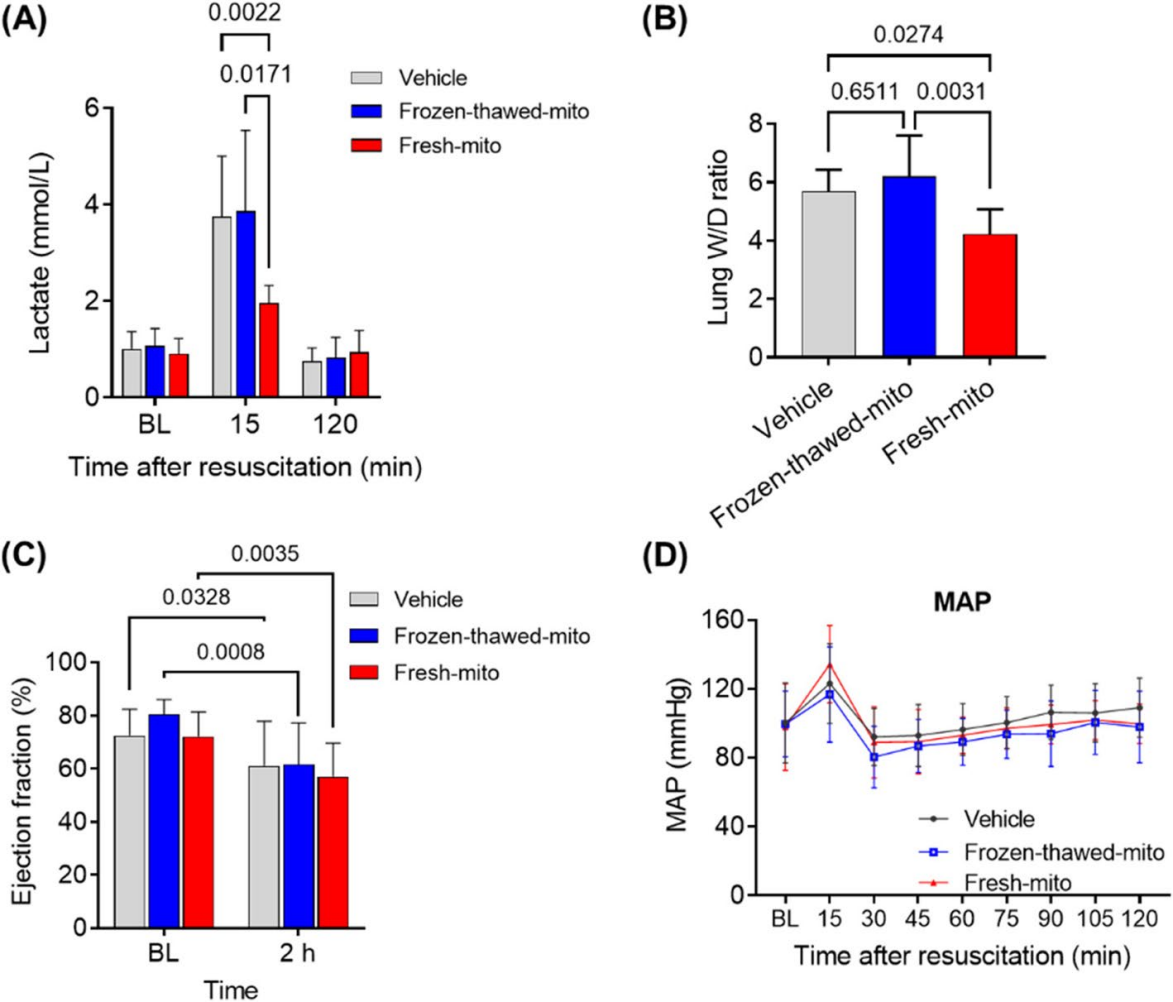
Mitochondrial transplantation enhances early lactate normalization and mitigates lung injury after cardiac arrest and resuscitation. **A** Arterial lactate levels at pre-arrest baseline and 15- and 120-min post-resuscitation. *n* = 11 per group. A mixed-effects model for repeated-measures analyses, followed by one-way analysis of variance (ANOVA) with Šidák’s correction for post hoc comparisons, was used. **B** Cardiac arrest-induced lung edema at 72 h post-resuscitation was diminished by delivering freshly isolated mitochondria. **C** Left ventricular ejection fraction at pre-arrest baseline and 2 h post-resuscitation in post-arrest rats treated with vehicle, frozen-thawed mitochondria (frozen-thawed-mito), or freshly isolated mitochondria (fresh-mito). A mixed-effects model for repeated-measures analysis, followed by ANOVA with Šidák’s correction for post hoc comparisons, was employed. **D** Changes in the mean arterial pressure (MAP). A mixed-effects model for repeated-measures analysis, followed by ANOVA with Šidák’s correction for post hoc comparisons, was used. Data represent the mean ± standard deviation

**Fig. 5:**
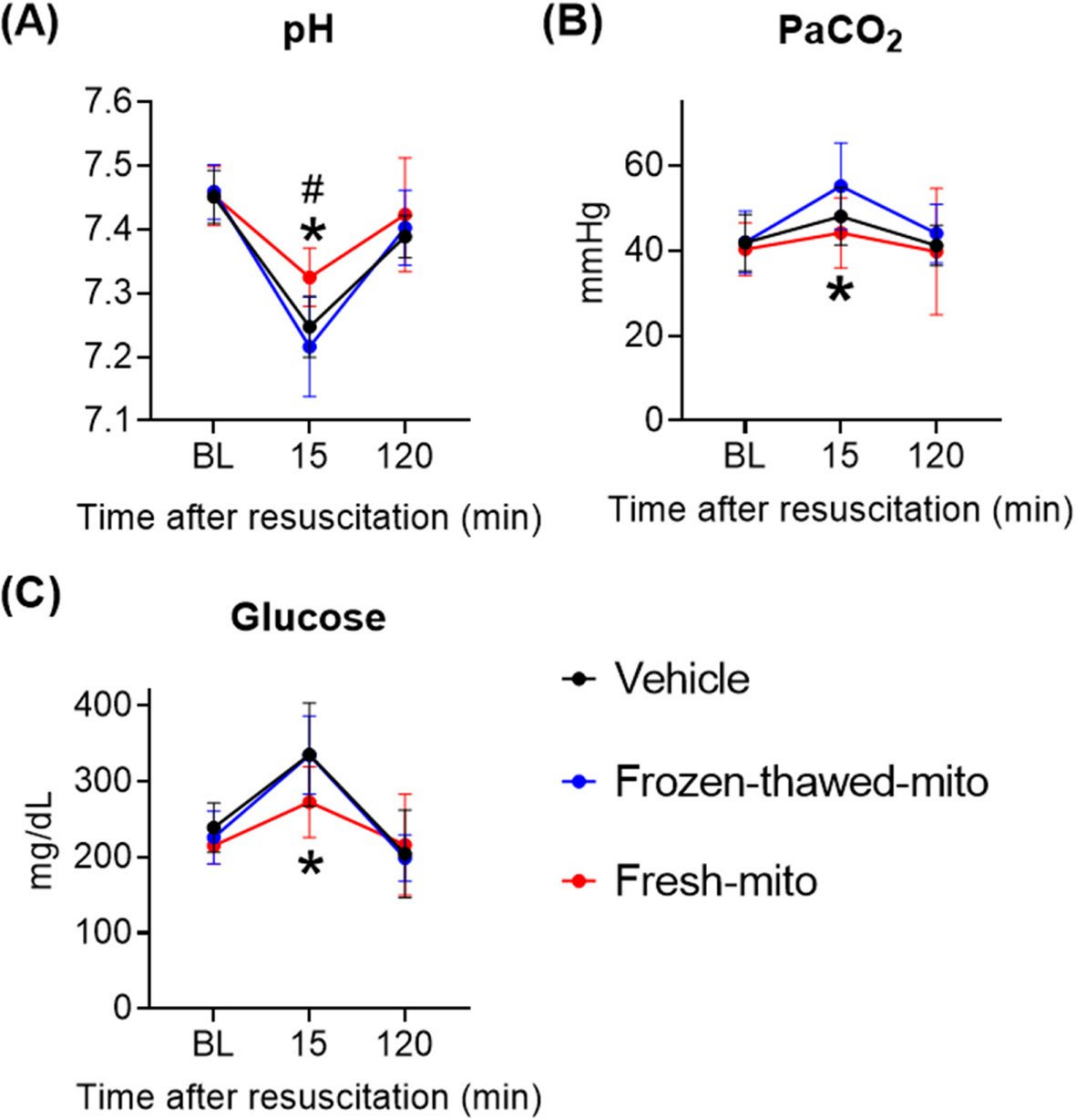
Mitochondrial transplantation normalizes metabolic parameters early after cardiac arrest and resuscitation. Arterial **A** pH, **B** partial pressure of carbon dioxide (PaCO_2_), and **C** glucose levels at pre-CA baseline and at 15 min and 2 h after resuscitation among groups. *P < 0.05; fresh-mito vs. vehicle group, ^#^*P* < 0.05; fresh-mito vs. frozen-thawed-mito group. *n* = 11 per group. A mixed-effects model for repeated-measures analysis, followed by ANOVA with Šidák’s correction for post hoc comparisons, was used. Data represent the mean ± standard deviation

Figure 4A shows the significant reduction of arterial lactate levels observed in post-CA rats within 15 min of resuscitation in the fresh-mito group compared to the higher lactate levels observed in the vehicle and frozen-thawed groups. We also assessed pulmonary edema at 72 h after resuscitation by measuring the water content of the lung, which was markedly lower in the fresh-mito group (W/D, 4.23 ± 0.85) relative to that in the vehicle (5.70 ± 0.75, *P* = 0.027) and frozen-thawed (6.21 ± 1.40, *P* = 0.003) groups (Fig. 4B). Echocardiography confirmed the lack of significant differences in early hemodynamic parameters. As expected, CA resulted in reduced LVEF at 2 h after resuscitation in all groups (Fig. 4C). However, we were unable to identify significant differences between the MTx and control groups for hemodynamic parameters of arterial pressure, heart rate, and left ventricular ejection fraction during the first 2 h of monitoring following resuscitation (Fig. 4C, D). Monitoring was discontinued after this time point; thus, any possible changes beyond this point could not be observed. Furthermore, we observed higher arterial pH at 15 min and lower arterial partial pressure of carbon dioxide in the fresh-mito group compared with the vehicle and frozen-thawed groups. Glucose levels, which are typically quite elevated in animals immediately after CA, were lower after 15 min in animals subjected to MTx compared with the other groups. Notably, these levels all returned to baseline at 120 min (Fig. 5). The groups did not differ in terms of partial pressure of oxygen, oxygen saturation, base excess, hematocrit, or blood electrolyte levels (Additional file 1: Fig. S1).

**Fig. 6:**
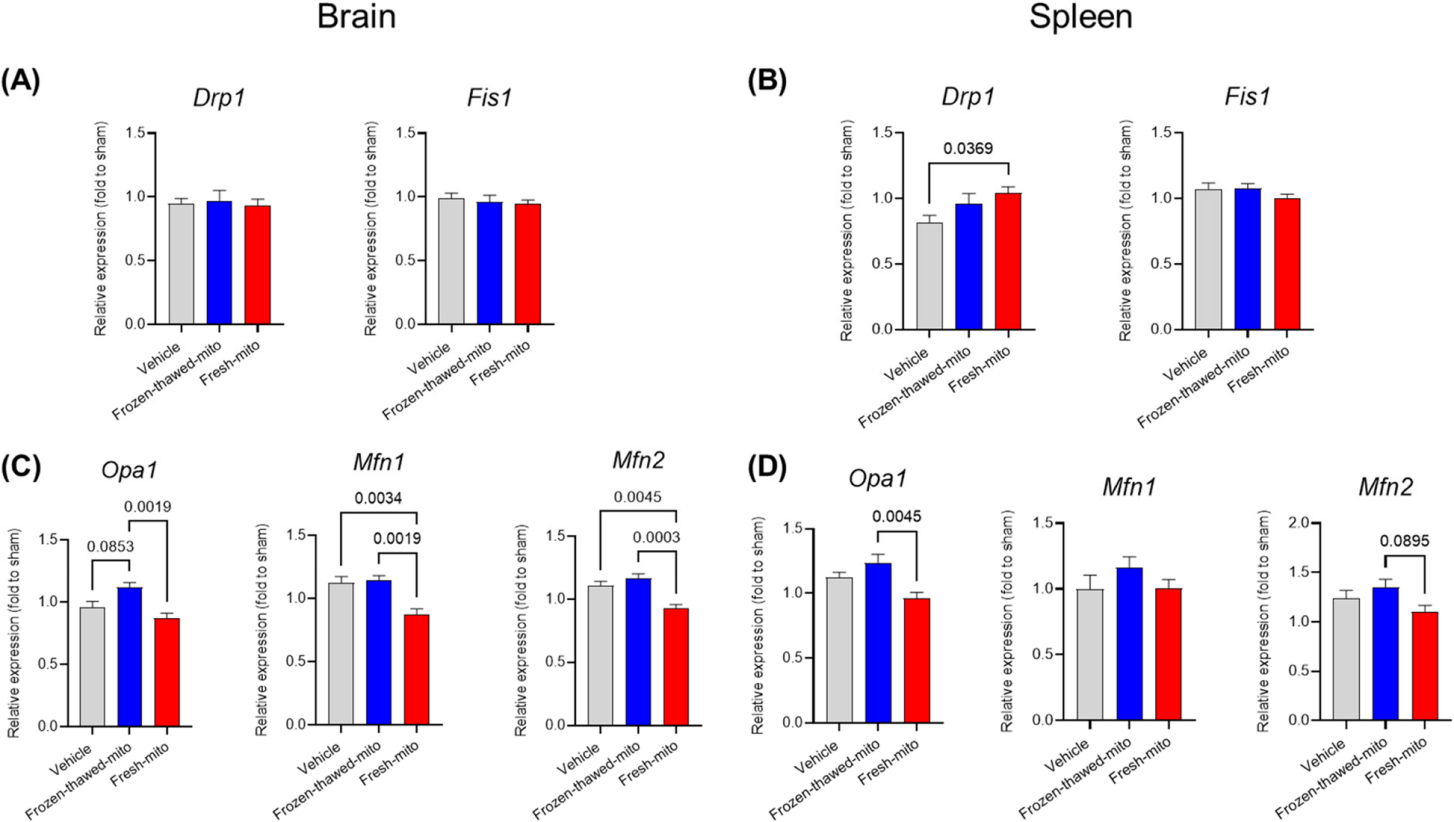
Gene expression changes in the brain and spleen in survived rats at 72 h after cardiac arrest and resuscitation with or without mitochondrial transplantation. Gene expression of molecules related with mitochondrial fission proteins (dynamin-related protein 1 [*Drp1*], mitochondrial fission 1 protein [*Fis1*]) in the **A** brain and **B** spleen and mitochondrial fusion proteins (optic atrophy-1 [*Opa1*], Mitofusin-1 [*Mfn-1*], Mitofusin-2 [*Mfn-2*]) in the **C** brain and **D** spleen. ANOVA with Sidak’s correction for post hoc comparisons was used. *n* = 6, 6, and 10 for the vehicle, frozen-thawed-mito, and fresh-mito groups, respectively. Data represent the mean ± standard deviation

To further characterize the mechanism of beneficial effects of fresh mitochondrial injection, we measured the changes in the gene expression of markers for mitochondrial fission (*Drp1*, *Fis1*) and mitochondrial fusion (*Opa1*, *Mfn1*, *Mfn2*) in tissue homogenates from the brain and spleen of sham-operated rats or surviving rats at 72 h post-CA in the vehicle, frozen-thawed, or fresh-mito group. The results are shown in Fig. 6. In the brain, fresh mitochondria markedly attenuated the gene expressions of all fusion proteins compared to frozen-thawed mitochondria. MTx with fresh mitochondria markedly decreased the gene expressions of *Mfn1* and *Mfn2* compared to the vehicle group, while frozen-thawed mitochondria did not affect the gene expressions of either fission or fusion proteins. The groups did not differ in terms of mitochondrial fission genes. In the spleen, fresh mitochondria markedly attenuated the gene expression of *Opa1* compared to frozen-thawed mitochondria. In addition, the *Drp1* gene expression in the spleen was markedly higher in the fresh-mito group than in the vehicle group.

We also measured the COX activity in tissue homogenates from the brain and spleen of sham-operated rats or surviving rats at 72 h post-CA in the vehicle, frozen-thawed, or fresh-mito group. The results are shown in Additional file 1: Fig. S2. The colorimetric enzymatic assay showed that the groups did not differ in terms of COX activity both in the brain and spleen. The COX activity in the vehicle group had no change compared to those in the sham-operation group, suggesting the recovery of COX activity in tissue homogenates 72 h after CA resuscitation in the surviving animals.

### MTx improved cerebral microperfusion early after CA and resuscitation

**Fig. 7:**
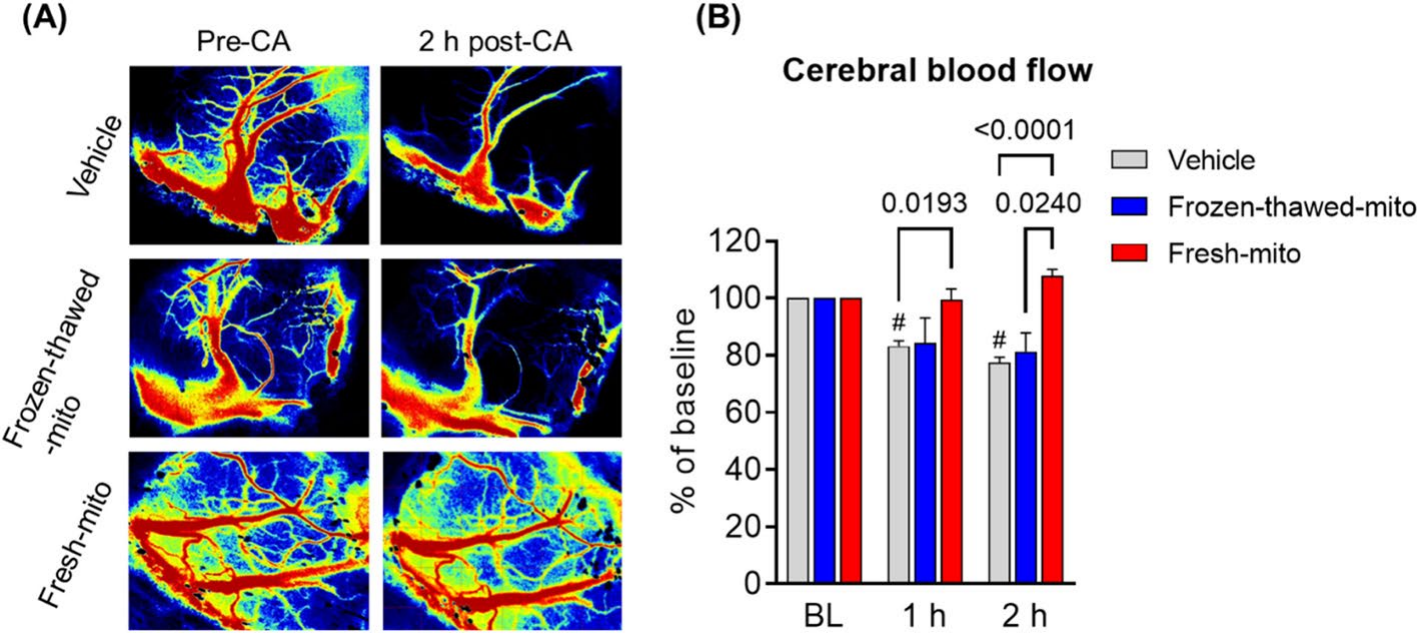
Mitochondrial transplantation enhances cerebral perfusion early after cardiac arrest and resuscitation. **A** Representative photographs of laser speckle contrast imaging at pre-CA baseline and at 2 h after CA in three groups. **B** Changes in the mean relative cerebral blood flow (rCBF) of ROIs in post-CA rats treated with vehicle, frozen-thawed mitochondria (frozen–thawed-mito), or freshly isolated mitochondria (fresh-mito). A mixed-effects model for repeated-measures analysis, followed by ANOVA with Šidák’s correction for post hoc comparisons, was used. *n* = 6 per group. Data represent the mean ± standard deviation

To further identify the effect of MTx on neurological function, we conducted a separate series of animal studies, measuring the impact of MTx on cerebral blood flow for 2 h following CA. We measured rCBF, where relative refers to baseline, in three ROIs on the cortical surface of the brain. We used a laser speckle imaging system, comparing rCBF measured minute by minute during CA until 2 h post-CA. Figure 7A shows representative images of the three ROIs from animals in each of the three groups at the baseline and 2 h after CA. Figure 7B compares the recovery of cerebral perfusion in the three groups (*n* = 6 in each group). Post-CA rats who received MTx had improved rCBF at 2 h compared to either control group: 107.7% ± 5.6% in the fresh-mito group compared to 77.5% ± 4.5% in the vehicle group (*P* < 0.0001) and 81.3% ± 15.9% in the frozen-thawed-mito group (*P* = 0.024).

### Persistence of transplanted mitochondria in the brain, kidney, and spleen at 24-h post-CA

**Fig. 8:**
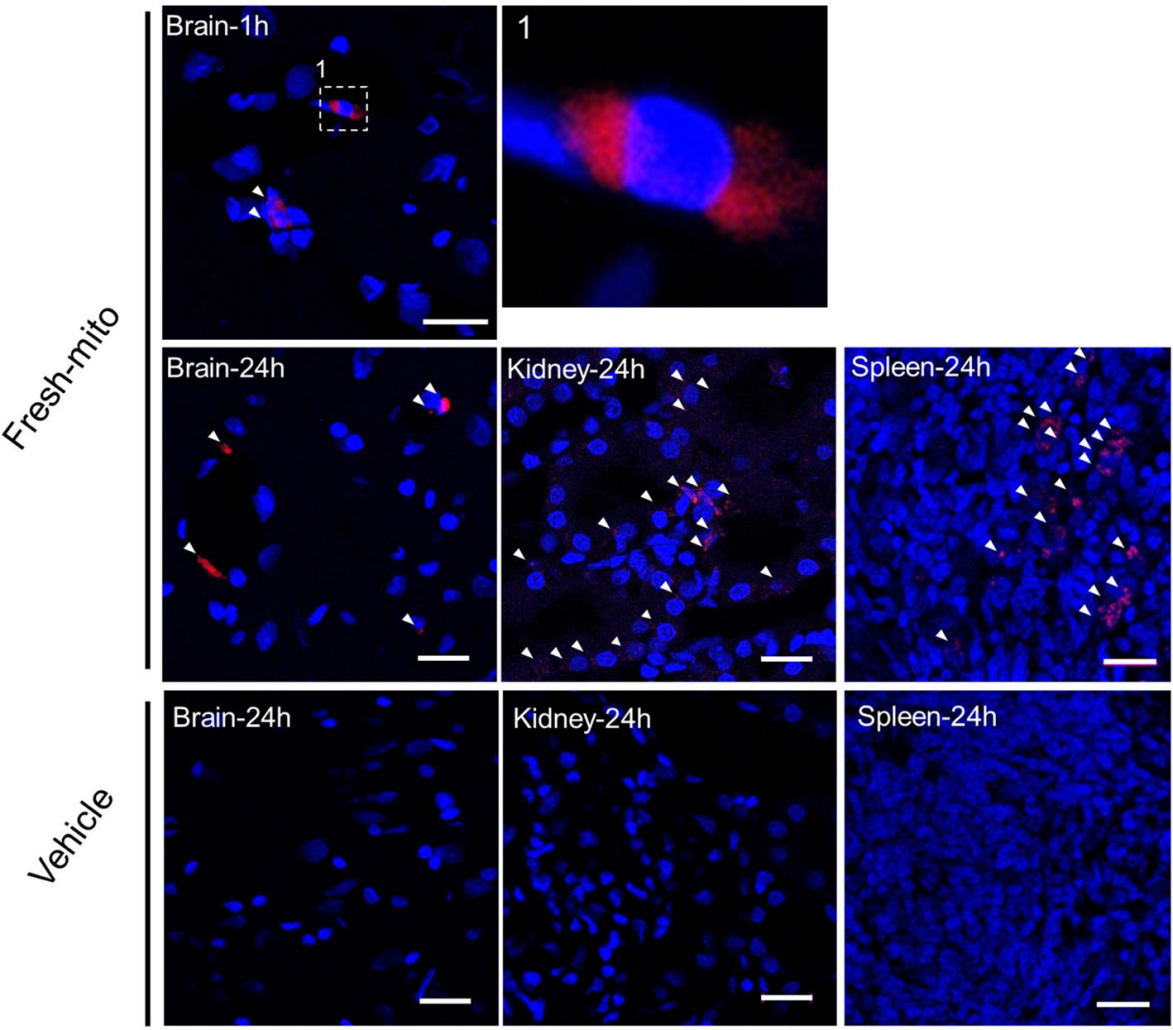
Confocal fluorescence imaging reveals the persistence of the donor mitochondria within the organs at 1 and 24 h after cardiac arrest and resuscitation. Transplanted mitochondria (red) were observed in the brain, kidney, and spleen at 1 and 24 h after cardiac arrest. Arrowheads indicate the donated mitochondrial particles labeled using MitoTracker Deep Red dye before the injection. The cell nuclei were counterstained with DAPI (blue). Scale bar indicates 20 µm

In an additional series of animal studies, we determined the visual persistence of labeled freshly isolated donor mitochondria within the critical organs and tissues of recipient CA animals via microscopy. Confocal fluorescence imaging of mitochondria labeled with MitoTracker Deep Red confirmed that at 1- and 24-h post-injection after CA, transplanted mitochondria were observed in the brain, kidney, and spleen (Fig. 8). We did not observe similar persistence of the labeled mitochondria after 24 h within the heart, liver, or lung (Additional file 1: Fig. S3).

## Discussion

In this paper, we report on the potential for MTx to mitigate the damage caused by the severe ischemic injury seen in CA. We found that MTx dramatically increased neurologically intact survival in a rat model where rats were resuscitated from 10 min of asphyxial CA. This improved survival was associated with improvements in metabolism, cerebral blood flow, and lung edema. Our in vitro studies confirm that neural cell cultures easily take up exogenous freshly isolated donor mitochondria and that donor mitochondria are capable of respiring and producing ATP. Follow-up in vivo studies determined that intravenously infused transplanted donor mitochondria can be found in tissues 24 h after CA.

Our primary finding was that MTx improved survival from 55 to 91% following CA. We were unable to find prior studies of MTx in the setting of CA; however, MTx has been reported to be protective in other animal disease models, such as cardiac I/R [10–17], stroke [6, 18], liver I/R [30, 31], kidney I/R [32, 33], lung I/R [25], spinal cord injury [34], Parkinson’s [35, 36], and schizophrenia [37]. For example, in a mouse stroke model, mitochondria derived from placental tissue were intravenously infused into animals following a focal carotid occlusion, and this resulted in a significant reduction in infarct size in 72 h [18]. In the ischemic brain, astrocytes may transfer healthy mitochondria into damaged neurons [6]. Another study found transplantation of muscle-derived mitochondria reduced cellular oxidative stress and apoptosis, decreased brain infarct volume, and reversed neurological deficits after ischemic stroke in rats [38].

We also verified that the frozen-thawed process markedly reduces ATP content and ΔψM in the isolated mitochondrial particles. This finding is important for two reasons: (1) it validates that our use of the rapid isolation procedure, reported by McCully et al., resulted in the extraction of functional ATP-generating donor mitochondria, and (2) freezing and thawing of mitochondria produced relatively nonfunctional mitochondria that are used for the subsequent series as a critical additional negative control group. Of note, most prior studies of MTx used the simple vehicle solution alone as the single negative control group [10–14, 16, 18, 25, 33, 35–37, 39]. For our MTx investigation, we wanted to confirm that it was the functional capacity of the infused mitochondria that is required for changes in outcome, and not due to infusion of another component present in nonfunctioning mitochondrial particles. Nonfunctional freeze-thawed mitochondrial particles contain similar quantities of mitochondrial membranes, proteins, and other macromolecules, and it is reasonable to believe that some of these components could have biological activity, perhaps acting as damage-associated molecular patterns or signaling molecules. The use of freeze-thawed mitochondria as an additional negative control allows us to eliminate an important possible confounder.

The translation of our findings to humans could occur rapidly, especially as some human trials using MTx have already been performed, others are ongoing, and more are anticipated. MTx has already been used in pediatric patients with myocardial ischemia post-cardiac surgery [39, 40]. Guariento et al. reported on pediatric patients with severe congenital heart disease on heart–lung machines who were injected with their own mitochondria. In their study, MTx was performed to speed up the weaning process and some degree of success was reported [39, 40]. Furthermore, a human study is in progress to investigate the safety of autologous MTx delivered to the brain during cerebral ischemia. Walker et al. biopsied a patient’s muscle tissue to isolate mitochondria at the bedside and infused the freshly isolated mitochondria directly into the patient’s brain (NCT04998357). We hope that the series of studies presented here will further stimulate research in this field.

In our study, we demonstrated that exogenous mitochondria migrate in culture media and co-localize with endogenous mitochondria in neural cell culture through simple co-culture for 24 h. The ability of mitochondria to migrate from one cell to another is a recent discovery and is currently a poorly understood phenomenon. Our results confirm that mitochondria easily transfer across the cell membrane; this may be a naturally occurring event that takes place more often than previously appreciated. Various studies support our in vitro findings. Andrabi et al. demonstrated the transfer of mitochondria from astrocytes and microglia into damaged neurons [41]. Hayakawa et al. found that mitochondria released into the extracellular space can transfer from cell to cell in the brain after a stroke. These findings suggest a new mitochondrial mechanism of neuroglial crosstalk that may contribute to endogenous neuroprotection after brain I/R injury [6, 18]. Spees et al. demonstrated the intercellular transfer of normal mitochondria from mesenchymal stem cells to mammalian cells harboring dysfunctional mitochondria [42]. Thus, mitochondria are increasingly considered to play an important role in cell-to-cell communication and function [41–44], and the transfer of mitochondria from healthy cells to damaged cells appears to be a promising therapeutic approach [6, 8, 9]. Further studies are needed to determine the precise mechanisms by which exogenous mitochondria are taken up by cells during I/R and to elucidate how extracellular mitochondria maintain their viability, migrate into tissues, and act to protect tissues during the transplantation process.

Our results of gene expression measurements suggest that mitochondrial dynamics are shifted toward fission in the fresh-mito group during a recovery phase in surviving animals resuscitated from CA, which might be associated with the beneficial effects of fresh mitochondria supplementation. It has been shown that inhibition of mitochondrial fission by Drp1 inhibitor provided beneficial effects against brain injury after global ischemia and reperfusion [45]. In contrast, a previous study demonstrated that inhibition of fission by inhibiting Drp1 or siRNA contributed to increasing damaged mitochondria-mediated injury such as ROS generation, cytochrome c release, and activation of caspase-3 after ischemic-hypoxic injury, aggravating the ischemic brain damage [46]. It has been demonstrated that mitochondrial fission enables mitochondria to segregate dysfunctional mitochondria which contain damaged protein, mutated DNA, or destabilized membranes [47]. These conflicting results suggest that the balance between fission and fusion and its role in brain recovery have not been elucidated yet. Moreover, the role of mitochondrial dynamics remains incompletely investigated not only for the acute phase but also for the late recovery phase from ischemia–reperfusion injury. Further investigations are warranted to elucidate the role of MTx on mitochondrial dynamics and brain injury after CA resuscitation.

An important finding in our study is the persistence of fluorescent-labeled mitochondria in critical tissues of animals at 24 h after CA. To date, limited literature exists on the anatomical location where circulating donor mitochondria are taken up following intravenous infusion. A number of prior studies have investigated the direct injection of mitochondria into the heart muscle and intracoronary injection. Those studies demonstrated the retention of donor mitochondria within the ischemic areas of the heart but not in other organs [10, 11, 16]. In contrast, a study by Nakamura et al., using the same intravenous infusion we performed, demonstrated that in addition to finding mitochondria in the ischemic brain regions after a stroke, infused mitochondria were also identified in various peripheral organs including the lung, liver, kidney, and heart [18]. A recent study by Shi et al. demonstrated functional improvements in Parkinson’s disease as a result of MTx. Their study determined the widespread distribution of intravenously infused exogenous mitochondria in many different tissues including the brain, liver, kidney, muscle, and heart and speculated that Parkinson’s disease may be in part caused by the multiple organ sequelae of mitochondrial disease [36]. While we identified the uptake of exogenous donor mitochondria in multiple organs at 24 h after CA, the mechanism for cellular uptake of infused mitochondria remains unknown. It has been suggested that exogenous mitochondria might enter via cellular endocytosis pathways [48–50]. Further studies are warranted to determine the transmembrane uptake mechanisms, define which tissues and cells take up the most infused mitochondria, and further delineate the timeline for the persistence of transplanted mitochondria within targeted tissues.

Unique to our study is the use of nonfunctional frozen-thawed mitochondria as an additional negative control. These frozen-thawed mitochondria did not have any of the protective effects seen with freshly isolated mitochondria. This suggests requirements for relatively healthy respiring and ATP-producing mitochondria for successful transplantation. Other studies have demonstrated that MTx can improve ATP levels [51], protect mitochondrial peptides, prevent excessive proinflammatory responses [52], and reduce cell death pathway activation [53]. Nevertheless, many important questions remain.

The preliminary studies presented here have a number of limitations. The precise mechanism by which MTx improved survival outcomes remains unknown. We have yet to ascertain whether ATP levels in recipient tissues are altered with MTx. We did not quantify the number of mitochondria taken up, nor did we determine whether the tissues that take up mitochondria were the tissues most responsible for improved outcomes. Furthermore, our dosage and timing of MTx may not be optimal. Our animal CA model is a relatively short-term 72-h model; hence, longer-term benefits (or adverse effects) remain unknown. Many of our measurements were taken at specific time points; thus, we may have failed to detect significant changes that occur at other time points. For translation into human therapies, additional studies are required to elucidate the optimal dose of donor mitochondria, the optimal isolation methods, and the ideal timing of MTx to treat I/R injuries. In addition, we focused these studies on allogeneic MTx; however, this may not be feasible in the acute clinical setting. For the development of pragmatic human therapies, the possibility of xenotransplantation, as opposed to allogeneic or autologous transplantation has been suggested as a viable future direction [11, 54, 55].

## Conclusions

We found that MTx performed immediately after resuscitation from CA improved survival and neurological recovery in rats. MTx was associated with rapid recovery of lactate, pH, and glucose levels; improved microcirculation and cerebral perfusion; and decreased lung injury. These results provide a foundation for future studies to enhance our basic understanding of mitochondrial biology and promote the development of MTx as a novel therapeutic strategy to save lives currently lost after CA.

## Supplementary Information

**Additional file 1: Fig. S1.** Arterial blood and metabolic measures sampled at pre-arrest baseline and at 15- and 120-min after resuscitation. **Fig. S2.** The cytochrome c oxidase (COX) activity in tissue homogenates from the brain and spleen of surviving animals at 72 h post-CA in the vehicle, frozen-thawed-, or fresh-mito group. **Fig. S3.** Confocal fluorescence imaging for the heart, liver, and lung at 24 h after CA resuscitation in rats treated with vehicle or fresh mitochondrial transplantation.

## Data Availability

The datasets used and/or analyzed during the current study are available from the corresponding author upon reasonable request.

## Abbreviations

MTx: Mitochondrial transplantation
CA: Cardiac arrest
ATP: Adenosine triphosphate
I/R: Ischemia and reperfusion
PBS: Phosphate-buffered saline
BSA: Bovine serum albumin
ΔψM: Mitochondrial membrane potential
CCCP: Carbonyl cyanide 3-chlorophenylhydrazone
RR: Respiratory rate
TV: Tidal volume
NFS: Neurological function score
LVEF: Left ventricular ejection fraction
W/D: Wet-to-dry weight ratio
Drp1: Dynamin-related protein 1
Fis1: Mitochondrial fission 1 protein
Opa1: Optic atrophy-1
Mfn-1: Mitofusin-1
Mfn-2: Mitofusin-2
COX: Cytochrome c oxidase
rCBF: Relative cerebral blood flow
ROI: Regions of interest
DAPI: 4,6-Diamidino-2-phenylindole
ANOVA: Analysis of variance
