# Supplementary material for: Exogenous mitochondrial transplantation improves survival and neurological outcomes after resuscitation from cardiac arrest
Source: BMC Med. 2023 Mar 16;21:56. doi: 10.1186/s12916-023-02759-0 (PMC10018842; doi:10.1186/s12916-023-02759-0)
Supplement: Supplementary file 1 — Additional file 1: Fig. S1. Arterial blood and metabolic measures sampled at pre-arrest baseline and at 15- and 120-min after resuscitation. Fig. S2. The cytochrome c oxidase (COX) activity in tissue homogenates from the brain and spleen of surviving animals at 72 h post-CA in the vehicle, frozen-thawed-, or fresh-mito group. Fig. S3. Confocal fluorescence imaging for the heart, liver, and lung at 24 h after CA resuscitation in rats treated with vehicle or fresh mitochondrial transplantation. [file 12916_2023_2759_MOESM1_ESM.docx]

**Additional file 1****: Figure S1-3**

**Figure S1.** **Arterial blood and metabolic measures sampled at pre-arrest baseline and at 15- and 120-min after resuscitation.**

A mixed-effects model for repeated-measures analysis, followed by analysis of variance (ANOVA) with Šidák’s correction for post hoc comparisons was used. Data represent the mean ± standard deviation.

**Figure S2.** **The cytochrome c oxidase (COX) activity in tissue homogenates from the brain and spleen of surviving animals at 72 h post-CA in the vehicle, frozen-thawed-, or fresh-mito group.**

The groups did not differ in terms of COX activity both in brain and spleen. ANOVA with Šidák’s correction for post hoc comparisons were used. n = 6, 6, 10 for the vehicle, frozen-thawed-mito, and fresh-mito groups, respectively. Data represent the mean ± standard deviation.

**
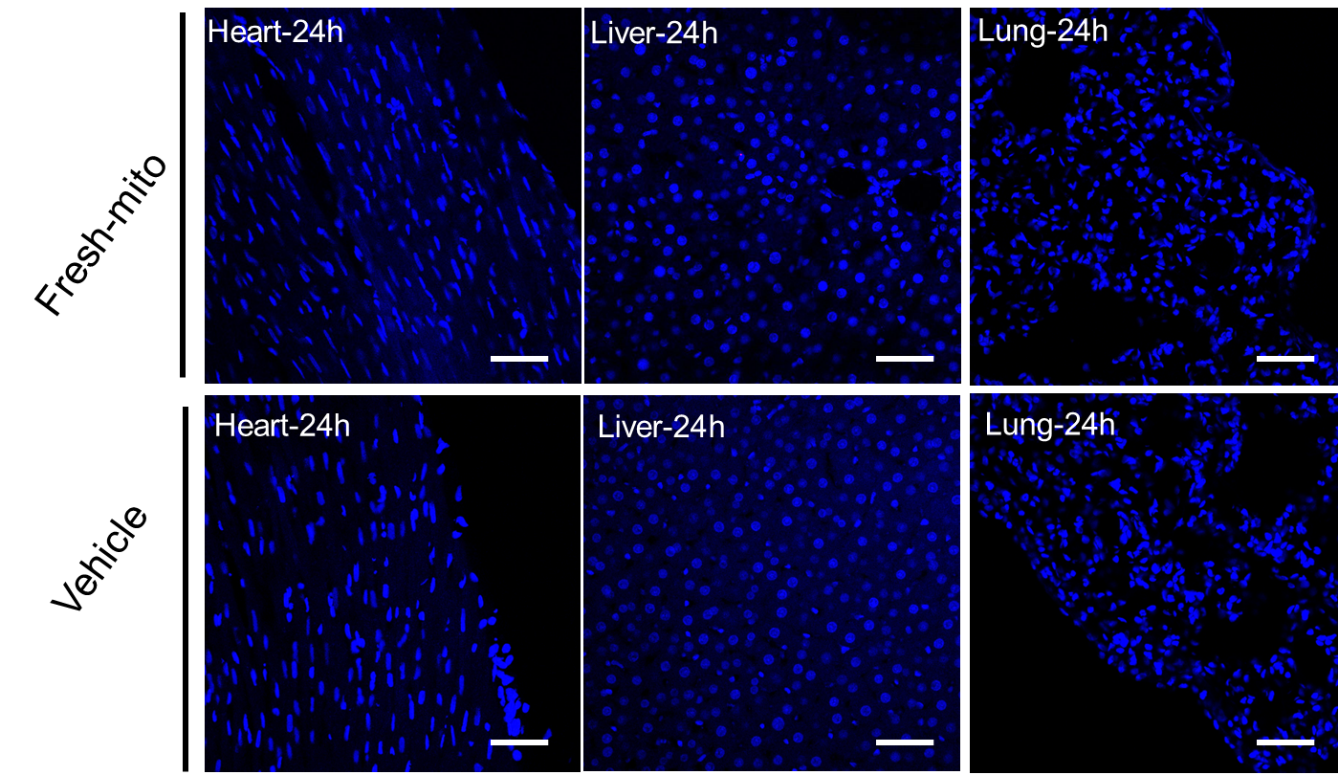
Figure S3. Confocal fluorescence imaging for the heart, liver, and lung at 24 h after CA resuscitation in rats treated with vehicle or fresh mitochondrial transplantation.**

The labeled mitochondria were not observed within the heart, liver, or lung in both groups.
